# Predictive value of serum β-hCG and blastocyst transfer for clinical pregnancy after frozen-thawed embryo transfer: a retrospective cohort study

**DOI:** 10.1186/s12884-026-09773-z

**Published:** 2026-08-05

**Authors:** Yun Le, Sheng Yang, Mengyi Zhu, Tengfei Wang, Guosong Shen, Yuanping Zhou, Lijun Peng

**Affiliations:** Department of Assisted Reproduction, Huzhou Maternity & Child Health Care Hospital, Huzhou, 313000 Zhejiang China

**Keywords:** Frozen-thawed embryo transfer, Human chorionic gonadotropin, Blastocyst transfer, Clinical pregnancy, Total protein

## Abstract

**Aim:**

To evaluate the predictive value of serum beta-human chorionic gonadotropin (β-hCG) measured at 11–14 days after frozen-thawed embryo transfer (FET) for clinical pregnancy, and to explore the potential associations of blastocyst transfer and other serum markers (total protein, albumin, red blood cell distribution width) with pregnancy outcomes.

**Methods:**

A retrospective cohort study was performed on 268 infertile patients who underwent FET at our hospital from January 2025 to December 2025. Patients were divided into clinical pregnancy (*n* = 132) and non-pregnancy (*n* = 136) groups. Baseline characteristics and serum markers (TP, Alb, RDW) measured on day 7 post-transfer were compared between groups. The predictive performance of β-hCG was evaluated using receiver operating characteristic (ROC) curve analysis. Univariate and multivariable logistic regression analyses were performed to identify factors independently associated with clinical pregnancy.

**Results:**

Serum β-hCG levels were significantly higher in the clinical pregnancy group (*P* < 0.001). ROC analysis showed that β-hCG predicted clinical pregnancy with an area under the curve (AUC) of 0.994 (95% CI: 0.989–0.999). The optimal cut-off value was 112.0 mIU/mL, yielding a sensitivity of 96.2% and a specificity of 98.5%. Multivariable logistic regression identified ln(hCG + 1) (OR = 12.55, 95% CI: 5.13–30.69, *P* < 0.001). Blastocyst transfer showed a trend towards an association in univariate analysis (OR = 1.59, 95% CI: 0.92–2.75, *P* = 0.10) but was not statistically significant in multivariable analysis (OR = 1.39, 95% CI: 0.30–6.32, *P* = 0.67). TP showed a trend towards an association in univariate analysis (OR = 0.95, 95% CI: 0.89–1.01, *P* = 0.10) but was not statistically significant, and was not an independent factor for clinical pregnancy (OR = 0.84, 95% CI: 0.68–1.03, *P* = 0.10). Alb and RDW showed no significant differences between groups.

**Conclusion:**

In this retrospective cohort study, serum β-hCG measured 11–14 days after FET demonstrated excellent predictive performance for clinical pregnancy (AUC 0.994). While both blastocyst transfer and total protein showed trends toward association in univariate analysis, neither remained an independent predictor in multivariable analysis. Other serum markers (Alb, RDW) provided no incremental predictive value beyond β-hCG. These findings support the clinical utility of early post-FET β-hCG measurement; however, the potential roles of blastocyst transfer and total protein require further investigation in larger, prospective cohorts.

With the continuous development of assisted reproductive technology (ART), frozen-thawed embryo transfer (FET) has become one of the core methods for the treatment of infertility. Compared with fresh embryo transfer, FET significantly reduces the risk of ovarian hyperstimulation syndrome (OHSS) while improving clinical safety by optimizing endometrial receptivity and embryo implantation timing [1, 2]. Nevertheless, the clinical pregnancy rate of FET is regulated by multiple factors including embryo quality, maternal metabolic status and immune function. How to accurately predict pregnancy outcomes and timely adjust treatment regimens remains an important issue in clinical practice.

## Serum β-hcg as a predictor of pregnancy outcomes

Serum β-hCG is a specific hormone secreted by trophoblast cells after embryo implantation. Its concentration directly reflects the quality of early embryo implantation and trophoblast proliferative activity, making it a core biomarker for early pregnancy assessment [3]. Previous studies have confirmed that early β-hCG levels following embryo transfer are closely associated with pregnancy outcomes.

 [4].However, differences exist among studies regarding the optimal cut-off value of β-hCG measured 11–14 days after FET, and its predictive value for adverse outcomes such as ectopic pregnancy and early miscarriage requires further validation.

 [5]. Moreover, relying solely on β-hCG may be insufficient to comprehensively assess pregnancy outcomes, as various factors—including embryo stage at transfer and maternal status—may jointly influence the implantation microenvironment [6, 7]. 

### The role of blastocyst transfer

Blastocyst transfer has been increasingly adopted in FET cycles due to its higher implantation potential. Studies have shown that blastocyst transfer improves clinical pregnancy and live birth rates [8, 9]. However, evidence regarding whether blastocyst transfer remains an independent predictor after adjusting for other factors, particularly β-hCG levels, is limited, with only a trend toward association reported in some studies.

### Exploratory serum markers: total protein, albumin, and RDW

In recent years, the regulatory effects of maternal systemic metabolism and inflammatory status on embryo implantation have attracted increasing attention.

 [10]. Serum total protein (TP) and albumin (Alb), as indicators of nutritional status, have been suggested to be potentially associated with FET outcomes, although direct evidence remains limited.Red cell distribution width (RDW) has been proposed as a surrogate marker for acute and chronic diseases and may be influenced by nutritional status, and also has prognostic value in various clinical conditions [11]. However, the predictive value of these markers for pregnancy outcomes after FET remains to be fully elucidated.

### Study Aim

Against this background, this retrospective study aimed to: (1) evaluate the predictive value of serum β-hCG measured at 11–14 days after FET for clinical pregnancy; (2) assess the association of blastocyst transfer with pregnancy outcomes; and (3) explore the potential associations of serum TP, Alb, and RDW measured on day 7 after transfer with clinical pregnancy. Receiver operating characteristic (ROC) curve analysis and multivariable logistic regression were performed to identify independent factors associated with pregnancy outcomes.

## Materials and methods

### Study subjects

This retrospective cohort study included FET cycles performed from January 1 to December 31,2025.Follow-up to 12 weeks of gestation was completed by March 31,2026. After data anonymization, the study was approved by the Ethics Committee of Huzhou Maternity & Child Health Care Hospital(Approval No. 2026-J-026) on April 2,2026,which waived written informed consent in accordance with national regulations and institutional guidelines. However, all patients had signed a general informed consent form before FET treatment, which included permission to use their anonymized data for scientific research.

#### Inclusion criteria

(1) Aged 20–40 years, with regular menstrual cycles (28–35 days), and no history of menstrual disorders or hormone-related medication use within the past 3 months; (2) Endometrial preparation using natural cycle, hormone replacement cycle, or mild stimulation cycle, with endometrial thickness ≥ 8 mm, endometrial pattern type A or B, and no abnormalities such as polyps or intrauterine fluid; (3) Transferred embryos were either day 3 cleavage-stage embryos or day 5 blastocysts; (4) No history of severe liver or kidney dysfunction, cardiovascular disease, thyroid dysfunction, diabetes mellitus, or other metabolic diseases; (5) No history of autoimmune diseases such as rheumatoid arthritis or systemic lupus erythematosus, and no history of chronic inflammatory diseases such as chronic pelvic inflammatory disease or ulcerative colitis; (6) No use of immunosuppressants, glucocorticoids, non-steroidal anti-inflammatory drugs (NSAIDs), or other medications affecting inflammatory cytokine levels or reproductive endocrinology within 3 months prior to embryo transfer; (7) No history of adverse lifestyle habits (e.g., smoking, alcohol abuse, drug use), with complete follow-up data (up to 12 weeks of gestation, no missing key test indicators or pregnancy outcome records).

#### Exclusion criteria

(1) Presence of severe systemic diseases such as severe liver or kidney dysfunction, cardiovascular and cerebrovascular diseases, or malignant tumors; (2) Uncontrolled endocrine or immune disorders, including hyperthyroidism/hypothyroidism, diabetes mellitus, or autoimmune diseases; (3) Use of glucocorticoids, immunosuppressants, or other medications that may affect inflammatory and metabolic indicators within the past 3 months; (4) Presence of uterine organic lesions (e.g., uterine malformations, intrauterine adhesions, stage III–IV endometriosis, adenomyosis), or a history of uterine surgery (e.g., myomectomy, intrauterine adhesion separation) within less than 6 months prior to the study; (5) History of malignant tumors or recent receipt of radiotherapy or chemotherapy; (6) Development of severe complications after embryo transfer (e.g., severe ovarian hyperstimulation syndrome, severe infection) affecting indicator testing or follow-up; (7) Incomplete clinical data, missing key test data, loss to follow-up, or inability to determine pregnancy outcomes.

### Methods

#### Embryo transfer and sample collection

All patients received routine FET clinical management. Endometrial preparation was performed via natural cycle, hormone replacement cycle or or other cycles (e.g., mild stimulation cycle).The optimal timing of embryo transfer was determined according to endometrial development, and qualified frozen-thawed embryos were transferred subsequently.

#### Outcome measures and variables

The primary outcome was clinical pregnancy, defined as an intrauterine gestational sac with fetal heartbeat at 28–30 days after transfer. Biochemical pregnancy was defined as serum β-hCG > 25 mIU/mL at 11–14 days post-transfer without subsequent clinical pregnancy.

Primary predictors were serum β-hCG at 11–14 days post-transfer and blastocyst transfer (vs. cleavage-stage embryo transfer). Secondary exploratory variables included serum total protein (TP), albumin (Alb), and red blood cell distribution width (RDW) measured on day 7 post-transfer.

Embryological assessment for statistical analysis: Embryo quality was assessed by experienced embryologists using standard morphological criteria. For the purpose of statistical analysis, the original scores were converted into an ordinal variable using a simplified 3-point scale (1 = best, 2 = moderate, 3 = worst). This conversion was performed solely for regression modeling and does not replace the standard embryological grading system.

For blastocysts (preferred when available): Gardner scores AA, AB, BA → 1; BB → 2; BC, CB → 3. (CC-grade blastocysts were not transferred at our center and were excluded)

For cleavage-stage embryos (day 3,used only when no blastocyst was transferred):

Morphological grades I → 1; II → 2; III/IV → 3.

For cycles with mixed-stage transfer (cleavage + blastocyst):

The blastocyst grade was used for conversion, as blastocyst morphology better reflects developmental potential.

This simplified ordinal grading allowed embryo quality to be included as a covariate in multivariable regression while preserving clinical interpretability.

Post-thaw survival was recorded as full survival (> 90% cells intact), partial survival (50%–90%), or no survival (< 50%). All cycles achieved full survival. Preimplantation genetic testing (PGT) is not routinely performed at our center; therefore, euploid status data were not available.

Endometrial thickness was measured on the day of embryo transfer. Due to incomplete recording, data were missing for approximately 35% of patients.

#### Grouping

Patients were divided into two groups based on pregnancy outcomes:

##### Clinical pregnancy group

Clinical pregnancy confirmed by ultrasound at 28–30 days after transfer.

##### Non-pregnancy group

No pregnancy or biochemical pregnancy only. Biochemical pregnancy was defined as serum β-hCG > 25 mIU/mL at 11–14 days post-transfer without ultrasound confirmation. Completely non-pregnant patients had serum β-hCG levels below the lower detection limit (< 0.2 mIU/mL).

#### Statistical analysis

##### Descriptive analysis 

Normally distributed continuous variables were presented as mean±standard deviation (SD) and compared using independent-sample t-test. Non-normally distributed variables were presented as median (interquartile range, IQR) and compared using Mann-Whitney U test. Categorical variables were expressed as frequencies (percentages) and compared using chi-square (χ²) test.

##### ROC curve analysis 

The predictive performance of serum β-hCG was evaluated using receiver operating characteristic (ROC) curve analysis. The area under the curve (AUC), 95% confidence interval (CI), optimal cut-off value (determined by the Youden index), sensitivity, specificity, positive predictive value (PPV), and negative predictive value (NPV) were calculated.

##### Log transformation of β-hCG 

To correct for the skewed distribution of serum β-hCG and to enable clinically interpretable effect sizes, β-hCG values were log-transformed as ln(β-hCG + 1) for all analyses.

##### Logistic regression analysis 

Univariate logistic regression was performed first. Variables with *P* ≤ 0.10 in univariate analysis (ln(β-hCG + 1), TP, and blastocyst transfer) were entered into the multivariable logistic regression model. In addition, the model included established confounders, including age and BMI. Embryo quality and endometrial thickness had been pre-adjusted in sensitivity analyses and were not included in the main model. Results are presented as odds ratios (OR) with 95% confidence intervals (CI). Analyses of albumin (Alb) and red blood cell distribution width (RDW) were exploratory in nature; therefore, no adjustment for multiple comparisons was applied to these secondary analyses. Results should be interpreted with caution and require confirmation in future studies.

##### Combined prediction model 

A combined logistic regression model incorporating ln(β-hCG + 1) and TP was constructed to assess whether TP provides incremental predictive value beyond β-hCG alone. The area under the curve (AUC) of the combined model and that of ln(β-hCG + 1) alone were compared using the DeLong test.

All statistical tests were two-sided, and *P* < 0.05 was considered statistically significant. All analyses were performed using SPSS version 26.0.

## Results

### Baseline characteristics

A total of 268 infertile patients who underwent FET were included in this study, with 132 achieving clinical pregnancy and 136 not.The two groups were comparable in baseline characteristics, including age, BMI, infertility duration, AMH, blastocyst transfer rate, embryo quality, double embryo transfer, endometrial preparation protocol, infertility etiology, prior implantation failure, and endometrial thickness (all *P* > 0.05). Detailed baseline characteristics are presented in Table 1.

**Table 1:** Baseline characteristics of patients

Characteristic	Clinical pregnancy group (*n* = 132)	Non-pregnancy group (*n* = 136)	Statistical value	*P* value
General characteristics				
Age (years)	32.09 ± 4.49	32.46 ± 4.25	t =−0.70	0.49
BMI (kg/m²)	22.85 ± 3.29	22.48 ± 3.66	t = 0.88	0.41
Duration of infertility (years)	3.59 ± 2.83	3.40 ± 2.96	t = 0.53	0.91
AMH	4.44 ± 3.01	3.96 ± 3.21	t = 1.25	0.57
Embryo-related factors				
Blastocyst transfer, n (%)	103(78.0%)	94(69.1%)	χ² = 2.73	0.10
Embryo quality, n (%)			χ² = 1.03	0.60
best	63 (47.7%)	60 (44.1%)		
moderate	41(31.1%)	40(29.4%)		
worst	28 (21.2%)	36(26.5%)		
Double embryo transfer, n (%)	64 (48.5%)	54 (39.7%)	χ² = 2.10	0.15
post-thaw survival				
- Full survival (per patient), n (%)	132 (100%)	136 (100%)		
- Full survival rate (per embryo), %	196(100%)	190(100%)		
Endometrial factors				
Endometrial thickness (mm)*	8.86 ± 1.62 (*n* = 83)	8.51 ± 1.70 (*n* = 90)	t = 1.37	0.17
Endometrial preparation, n (%)			χ² = 5.89	0.053
- Natural cycle	24 (18.2%)	23 (16.9%)		
- Hormone replacement cycle	81 (61.4%)	99 (72.8%)		
- Mild stimulation cycle	27 (20.5%)	14 (10.3%)		
Infertility etiology, n (%)			χ² = 4.46	0.49
- Tubal factor	50 (37.9%)	64 (47.1%)		
- Male factor	7 (5.3%)	8 (5.9%)		
- Ovulatory disorder	23 (17.4%)	16 (11.8%)		
- Endometriosis	5 (3.8%)	7 (5.1%)		
- Unexplained	9 (6.8%)	11 (8.1%)		
- Mixed factors	38 (28.8%)	30 (22.1%)		
Prior implantation failure, n (%)			χ² = 1.81	0.18
- Yes	60 (45.5%)	73 (53.7%)		
- No	72 (54.5%)	63 (46.3%)		

Embryo quality was categorized as excellent, good and poor according to standard grading criteria for cleavage-stage embryos and blastocysts respectively

Embryo quality was graded on a 3-point scale: 1 = best (grade A for blastocysts or grade I for cleavage-stage embryos), 2 = moderate (grade B or grade II), 3 = worst (grade C or grade III/IV)

Post-thaw survival was recorded as full survival (> 90% of cells intact). All cycles achieved full survival. Preimplantation genetic testing (PGT) is not routinely performed at our center; therefore, euploid status data were not available

Endometrial thickness data were available for 173 patients (83 in the clinical pregnancy group and 90 in the non-pregnancy group) due to missing data (approximately 35%)

Embryo quality was converted into a 3-point ordinal scale (1 = best, 2 = moderate, 3 = worst) for statistical analysis based on original morphological assessments. Detailed conversion criteria are provided in the Methods Sect. (1.2.2)

*BMI* Body mass index, *AMH* Anti-Müllerian hormone

*Data are presented as mean ± standard deviation (SD), median (interquartile range, IQR), or number (percentage).

Bold P values indicate statistical significance (*P*< 0.05)

### Pregnancy outcomes

Pregnancy outcomes of both groups are summarized in Table 2. In the clinical pregnancy group (*n* = 132), 28 (21.2%) experienced miscarriage. By the data lock date (March 31, 2026), 72 (54.6%) had delivered live births, and the remaining had ongoing pregnancies (> 12 weeks of gestation). In the non-pregnancy group (*n* = 136), 31 (22.8%) had biochemical pregnancy, and 105 (77.2%) were completely non-pregnant. No patients were lost to follow-up. Due to the limited follow-up period (up to 12 weeks of gestation) and incomplete delivery data by the data lock date, complete live birth data were not available; therefore, clinical pregnancy was used as the primary outcome.Detailed baseline characteristics are presented in Table 1.

**Table 2:** Pregnancy Outcomes of patients

Group	Biochemical pregnancy	Clinical pregnancy	Miscarriage	Live birth*	Ongoing pregnancy	Lost to follow-up
Clinical pregnancy group (*n* = 132)	N/A	132(100%)	28(21.2%)	72(54.6%)	32(24.2%)	0(100%)
Non-pregnancy group (*n* = 136)	31(22.8%)	0(100%)	0(100%)	0(100%)	0(100%)	0(100%)

Ongoing pregnancy was defined as clinical pregnancy beyond 12 weeks of gestation without miscarriage by the data lock date. These patients had not yet delivered by the end of follow-up

*Live birth data were collected up to the data lock date (March 31, 2026). By this date, 72 patients in the clinical pregnancy group had delivered, while the remaining patients had not yet reached full term. Therefore, the live birth rate may be underestimated

### Comparison of serum markers between groups

Serum β-hCG levels at 11–14 days post-transfer were significantly higher in the clinical pregnancy group than in the non-pregnancy group (median: 700.5 vs. 40.1 mIU/mL, *P* < 0.001). And ln( hCG + 1) showed a similar trend.

(6.45 ± 0.99 vs. 1.03 ± 1.60, *P* < 0.001). Serum TP levels on day 7 post-transfer were slightly but significantly lower in the clinical pregnancy group compared with the non-pregnancy group (77.18 ± 4.22 vs. 77.98 ± 3.61 g/L, *P* = 0.04). No significant differences were observed in Alb or RDW between the two groups.

(both *P* > 0.05). Results are summarized in Table 3.

**Table 3:** Comparison of dserum markers between groups

Variable	Clinical pregnancy group (*n* = 132)	Non-pregnancy group (*n* = 136)	Statistical value	*P* value
**β-hCG (day 11–14 post-transfer**,** mIU/mL)**, median (IQR)*	700.5(427.0,1238.8)	40.1(26.0,76.2)	z = 9.52	< 0.001
**ln(β-hCG + 1) (day 11–14)**,** mean ± SD**	6.45 ± 0.99	1.03 ± 1.60	t = 33.26	< 0.001
**TP (day 7 post-transfer**,** g/L)**,** mean ± SD**	77.18 ± 4.22	77.98 ± 3.61	t =−1.65	0.04
**Alb (day 7 post-transfer**,** g/L)**,** mean ± SD**	44.06 ± 2.05	43.91 ± 2.10	t = 0.61	0.60
**RDW (day 7 post-transfer**,** %)**,** mean ± SD**	12.70 ± 0.92	12.81 ± 1.26	t =−0.79	0.32

*The non-pregnancy group included 31 biochemical pregnancies (β-hCG 25–100 mIU/mL) and 105 completely non-pregnant cases (β-hCG < 0.2 mIU/mL)

Bold P values indicate statistical significance (*P*< 0.05)

### Predictive value of serum β-hCG for clinical pregnancy

**Fig. 1:**
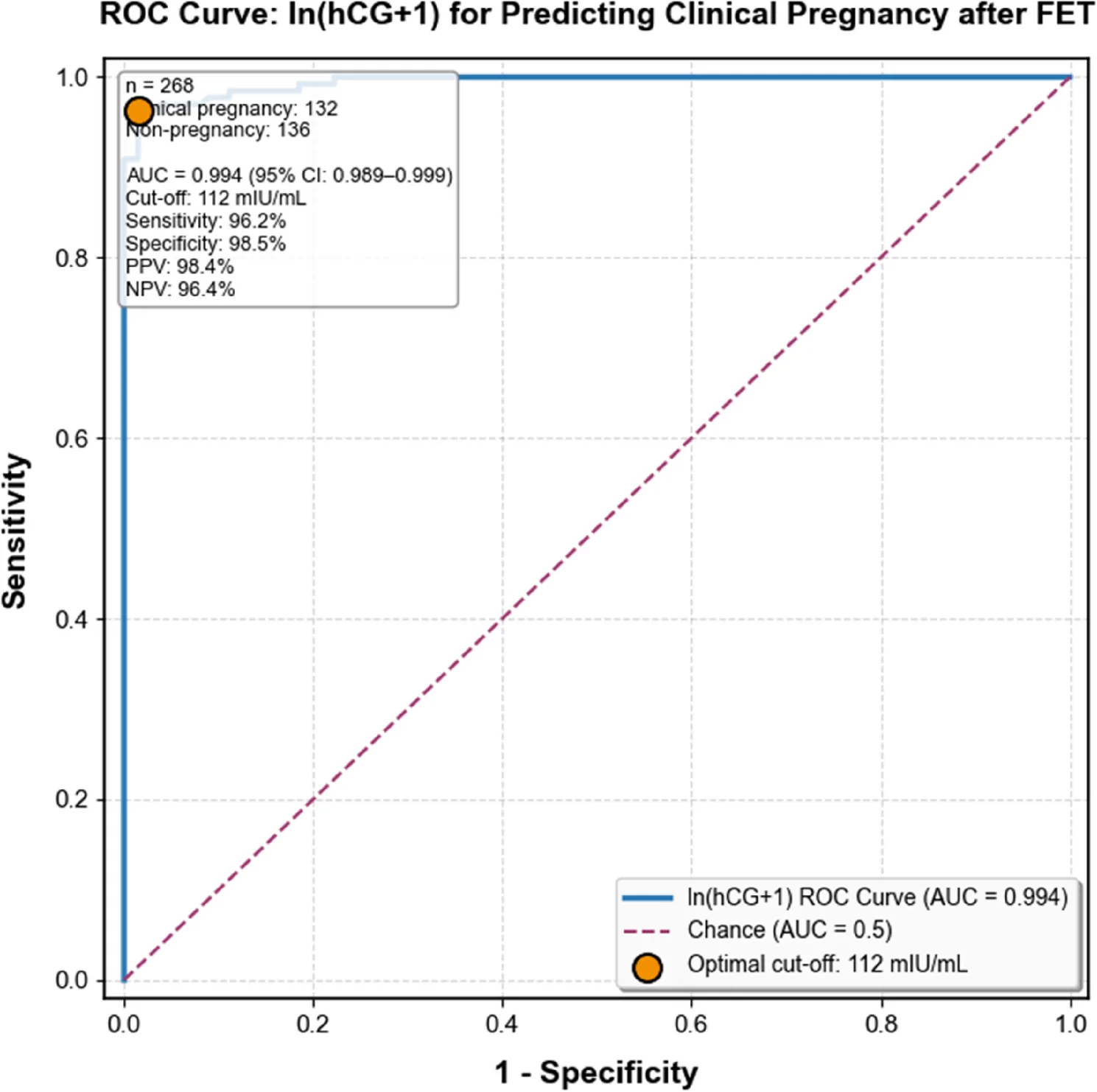
ROC curve of ln(β-hCG + 1) for predicting clinical pregnancy after frozen-thawed embryo transfer

ROC curve analysis based on ln(β-hCG + 1) was performed in all 268 patients, with clinical pregnancy (*n* = 132) as the positive outcome and non-clinical pregnancy (*n* = 136, including 31 biochemical pregnancies and 105 completely non-pregnant cases) as the negative outcome. Serum ln(β-hCG + 1) measured at 11–14 days after FET showed good predictive performance for clinical pregnancy, with an AUC of 0.994 (95% CI: 0.989–0.999, *P* < 0.001). The optimal cut-off value identified in our cohort was 112.0 mIU/mL, yielding a sensitivity of 96.2%, a specificity of 98.5%, a positive predictive value (PPV) of 98.4%, and a negative predictive value (NPV) of 96.4% (Fig. 1).

Figure 1. ROC curve of ln(β-hCG + 1) for predicting clinical pregnancy after frozen-thawed embryo transfer (*n* = 268). The area under the curve (AUC) was 0.994 (95% CI: 0.989–0.999, *P* < 0.001). The optimal cut-off value on the natural log scale was 4.72, corresponding to a raw β-hCG value of 112.0 mIU/mL after back-transformation (e^4.72–1). This cut-off yielded a sensitivity of 96.2%, a specificity of 98.5%, a positive predictive value (PPV) of 98.4%, and a negative predictive value (NPV) of 96.4%. The diagonal dashed line represents the reference line (AUC = 0.5).

### Univariate logistic regression analysis

Univariate logistic regression analysis showed that ln(β-hCG + 1) was significantly associated with clinical pregnancy (OR = 12.39, 95% CI: 5.16–29.73, *P* < 0.001). Based on a prespecified threshold of *P* < 0.10, TP (OR = 0.95, *P* = 0.10) and blastocyst transfer (OR = 1.59, *P* = 0.10) were included in the multivariable analysis. No other variables reached statistical significance (all *P* > 0.05). Detailed results are presented in Table 4.

**Table 4:** Univariate logistic regression analysis of factors associated with clinical pregnancy

Variable	OR (95% CI)	*P* value
**ln(β-hCG + 1)**	12.39 (5.16–29.73)	< 0.001
**TP (g/L)**	0.95(0.89–1.01)	0.10
**Blastocyst transfer (vs. cleavage-stage)**	1.59 (0.92–2.75)	0.10
**Double embryo transfer (vs. single)**	1.39 (0.86–2.25)	0.19
Embryo quality (per grade increase)*	0.87 (0.65–1.17)	0.37
**Endometrial thickness (per mm)**	1.14 (0.94–1.37)	0.18
**Prior implantation failure (vs. none)**	0.72 (0.45–1.16)	0.18
**Endometrial preparation(per category increase)**	1.31(0.86–2.00)	0.21
**Infertility etiology(per grade increase)**	1.08(0.97–1.22)	0.17

Endometrial preparation protocol was classified as: 1 = natural cycle, 2 = hormone replacement cycle, 3 = mild stimulation cycle

Infertility etiology was classified as: 1 = tubal factor, 2 = male factor, 3 = ovulatory disorder, 4 = endometriosis, 5 = unexplained, 6 = combined factors

*Embryo quality was graded on a 3-point scale: 1 = best (grade A for blastocysts or grade I for cleavage-stage embryos), 2 = moderate (grade B or grade II), 3 = worst (grade C or grade III/IV)

Bold P values indicate statistical significance (*P*< 0.05)

### Multivariable logistic regression analysis

Multivariable logistic regression was performed to identify factors independently associated with clinical pregnancy. Model 1 included ln(β-hCG + 1), age, and BMI as covariates. As shown in Table 5, ln(β-hCG + 1) was significantly associated with clinical pregnancy (OR = 12.55, 95% CI: 5.13–30.69, *P* < 0.001), while age and BMI showed no significant associations.

Model 2 additionally included blastocyst transfer and TP based on the prespecified threshold (*P* < 0.10) from univariate analysis. As shown in Table 6, ln(β-hCG + 1) remained significantly associated with clinical pregnancy (OR = 16.42, 95% CI: 5.63–47.90, *P* < 0.001), while blastocyst transfer (OR = 1.39, *P* = 0.67) and TP (OR = 0.84, *P* = 0.10) were not statistically significant.

**Table 5:** Multivariable logistic regression analysis of factors associated with clinical pregnancy (Model 1)

Variable	OR (95% CI)	*P* value
ln(β-hCG + 1)*	12.55 (5.13–30.69)	< 0.001
Age (years)	1.07 (0.89–1.27)	0.48
BMI (kg/m²)	1.04 (0.84–1.30)	0.70

**Table 6:** Multivariable logistic regression analysis of factors associated with clinical pregnancy (Model 2, extended)

Variable	OR (95% CI)	*P* value
ln(β-hCG + 1)*	16.42(5.63–47.90)	< 0.001
**Age (years)**	1.08 (0.90–1.30)	0.39
**BMI (kg/m²)**	1.09 (0.86–1.39)	0.47
**Blastocyst transfer (vs. cleavage-stage)**	1.39 (0.30–6.32)	0.67
**TP (g/L)**	0.84(0.68–1.03)	0.10

*ln(β-hCG + 1): natural logarithm of (β-hCG + 1)

Bold P values indicate statistical significance (*P* < 0.05)

### Sensitivity analysis

To assess the potential impact of embryo quality on our findings, we added embryo quality (graded 1–3) as an ordinal covariate into the extended multivariable model. After adjusting for embryo quality, ln(β-hCG + 1) remained significantly associated with clinical pregnancy (OR = 17.62, 95% CI: 5.65–55.90, *P* < 0.001), comparable to the main model result (OR = 16.42). Embryo quality itself was not significantly associated with clinical pregnancy (grade 2 vs. grade 1: OR = 0.56, *P* = 0.58; grade 3 vs. grade 1: OR = 0.80, *P* = 0.84). These findings confirm that excluding embryo quality from the primary model did not substantially bias our main conclusions.

A sensitivity analysis adding endometrial thickness was precluded due to substantial missing data (35%) and resultant model instability; thus, only the primary model without endometrial thickness is presented.

### Combined prediction model

**Fig. 2:**
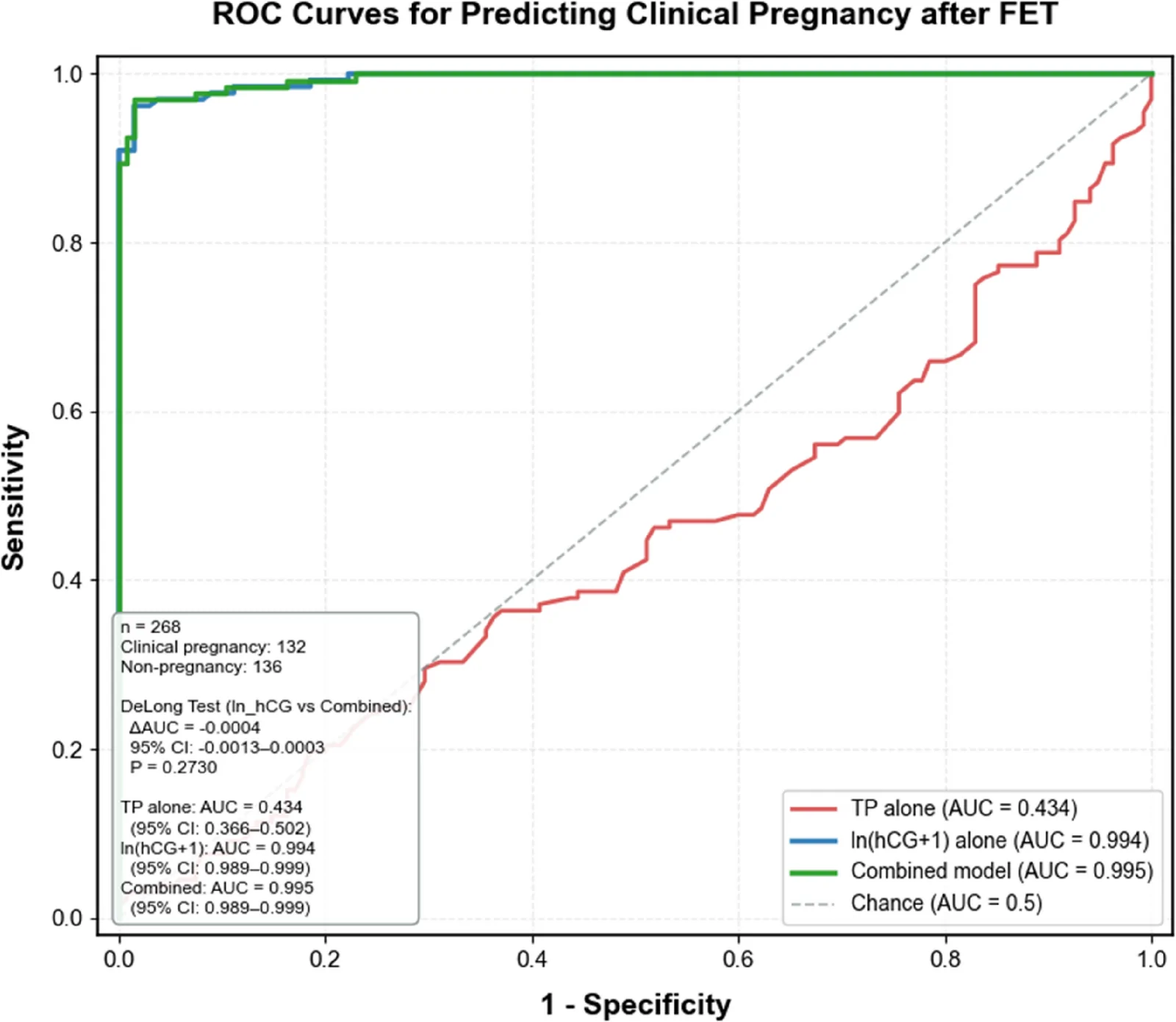
ROC curves of TP alone, ln(β-hCG + 1) alone, and the combined model for predicting clinical pregnancy after FET

A combined logistic regression model incorporating ln(β-hCG + 1) and TP was constructed to assess whether TP provides additional predictive value beyond β-hCG alone. The AUC of the combined model was 0.995 (95% CI: 0.989–0.999), compared with 0.994 (95% CI: 0.989–0.999) for ln(β-hCG + 1) alone. TP alone showed poor predictive performance (AUC = 0.434,95% CI: 0.366–0.502).The difference between the combined model and β-hCG alone was not statistically significant (DeLong test, *P* > 0.05), indicating that adding TP did not provide significant incremental predictive value (Fig. 2).

Figure 2. ROC curves of TP alone, ln(β-hCG + 1) alone, and the combined model for predicting clinical pregnancy after FET (*n* = 268). The AUC of ln(β-hCG + 1) alone was 0.994 (95% CI: 0.989–0.999). The AUC of the combined model (ln(β-hCG + 1) + TP) was 0.995 (95% CI: 0.989–0.999). TP alone showed poor predictive performance with an AUC of 0.434 (95% CI: 0.366–0.502). The diagonal dashed line represents the reference line (AUC = 0.5). The DeLong test showed no statistically significant difference between the combined model and ln(β-hCG + 1) alone (*P* > 0.05).

## Discussion

### Value of serum biomarkers in FET prognosis assessment

As a key component of ART, the pregnancy outcomes of FET are regulated by multiple factors, including embryo quality, endometrial receptivity, and the maternal systemic metabolic, endocrine, and immune status. Due to their convenience and quantifiability, serum biomarkers have become important tools for the early clinical prediction of FET pregnancy outcomes.Among these, the β-hCG level is a critical predictor of pregnancy outcomes following FET [12]. Alb and TP reflect nutritional status; however, in this study, total protein only showed a trend toward association with clinical pregnancy and did not reach statistical significance after multivariable adjustment. Blastocyst transfer also demonstrated only a trend toward association in this study and was not an independent predictor. These findings suggest that β-hCG has good clinical value for early pregnancy assessment after FET, while the potential roles of total protein and blastocyst transfer warrant further investigation.

### β-hCG: from post-implantation marker to clinical predictor

Serum β-hCG is a specific hormone secreted by trophoblast cells after embryo implantation. Its level is closely related to trophoblast proliferative activity and the quality of embryo implantation, making it a core indicator of early embryonic developmental potential and one of the gold standards for assessing pregnancy status [13]. However, it is important to recognize that β-hCG measured at 11–14 days after FET is a post-implantation biomarker that reflects established implantation and trophoblast function, rather than a pre-implantation predictor. Therefore, its predictive novelty is inherently limited.Numerous studies have confirmed that serum β-hCG levels measured 11–14 days after embryo transfer are significantly positively correlated with clinical pregnancy outcomes in FET: higher β-hCG levels are associated with higher implantation success and clinical pregnancy rates; conversely, low levels indicate poor trophoblast proliferation and increased risks of implantation failure or biochemical pregnancy [14]. In addition, the dynamic growth trend of β-hCG has important warning value for adverse pregnancy outcomes. Studies have shown that low or slowly rising β-hCG levels often suggest poor embryo implantation and are associated with significantly increased risks of ectopic pregnancy and early miscarriage [15].This finding is consistent with the 2022 study by Hernandez-Nieto et al. The trophectoderm serves as the morphological basis for β-hCG secretion by syncytiotrophoblasts after embryo implantation. Embryos with higher trophectoderm grades possess a thicker syncytiotrophoblast layer and greater cellular activity, thereby demonstrating a stronger capacity for β-hCG synthesis and secretion. Based on this mechanism, a high β-hCG level not only indicates successful embryo implantation but also suggests enhanced trophoblast function and greater potential for pregnancy maintenance. However, their study was limited to euploid embryo transfer cycles, and the conclusions may not be directly generalizable to the conventional frozen-thawed embryo transfer population without genetic screening [16]. This study included consecutive frozen-thawed embryo transfer cycles without preimplantation genetic testing, and the results showed that the predictive performance of β-hCG was consistent with the trend observed in euploid cycles, suggesting that β-hCG, as a post-implantation biomarker, possesses cross-population stability and general applicability.

### Predictive performance and cut-Off value of β-hCG in this study

In this study, ROC curve analysis was performed including all 268 patients (132 clinical pregnancies and 136 non-clinical pregnancies, the latter comprising 31 biochemical pregnancies and 105 completely non-pregnant cases). The area under the curve (AUC) for serum β-hCG measured 11–14 days after transfer in predicting clinical pregnancy was 0.994 (95% CI: 0.989–0.999, *P* < 0.001), indicating good.

predictive performance. This result is consistent with the findings of Wang Tao et al. [13], who reported an AUC of 0.926 for day 14 serum β-hCG in predicting live birth after FET. The optimal cut-off value was 112.0 mIU/mL, yielding a sensitivity of 96.2% and a specificity of 98.5%.A study by Wu et al. [17]further confirmed that in natural cycle FET, the cut-off value for day 12 serum β-hCG in predicting clinical pregnancy in the blastocyst transfer group was 217.70 mIU/mL (AUC = 0.924).The lower cut-off identified in our study likely reflects the inclusion of completely non-pregnant patients (β-hCG < 0.2) in the negative control group. Multivariate logistic regression analysis further confirmed that serum ln(β-hCG + 1) is an independent predictor of clinical pregnancy in FET (OR = 12.55, 95% CI: 5.13–30.69, *P* < 0.001).

 [18]. After adjusting for age and BMI, β-hCG remained strongly associated with clinical pregnancy.

### Exploratory analysis of total protein, albumin, and RDW

TP, Alb, and Glb reflect nutritional status, metabolic level, and immune tolerance function. Decidualization of endometrial stromal cells is a critical step in establishing receptivity, for which an appropriate metabolic environment is essential. Studies have shown that the receptive endometrium exhibits a specific protein expression profile, suggesting that protein metabolism is closely related to endometrial receptivity [11]. Therefore, monitoring serum TP levels before and after embryo transfer may help assess nutritional status.

During pregnancy, the decrease in serum TP may be partly attributed to physiological hemodilution resulting from increased maternal blood volume. Additionally, since the embryo is a semi-allogeneic graft, the mother must initiate immune tolerance mechanisms to suppress abnormal immune responses [19]. Glb, as the main protein component of immune responses, undergoes adaptive downregulation, further contributing to TP reduction. This explains the core pathophysiological mechanism underlying lower TP levels in the clinical pregnancy group: the combined effect of hemodilution and immune-related globulin downregulation. In patients with biochemical pregnancy, due to unstable embryo implantation and poor trophoblast development, maternal circulatory remodeling and immune tolerance initiation are incomplete, resulting in a much smaller decrease in TP compared with the clinical pregnancy group. In this study, TP levels in the pregnancy group measured 7 days post-transfer were slightly but significantly lower than those in the non-pregnancy group (77.18 ± 4.22 vs. 77.98 ± 3.61 g/L, *P* = 0.04). However, in the combined model incorporating both β-hCG and TP, the AUC did not significantly improve compared with ln(β-hCG + 1) alone (0.995 vs. 0.994, *P* > 0.05), suggesting that TP does not provide meaningful incremental predictive value beyond β-hCG. There was no significant difference in Alb between groups, and RDW also showed no significant differences.Of note, the analysis of TP was exploratory in nature. The proposed mechanisms (hemodilution and immune tolerance) are speculative and not confirmed by our data. The marginal significance (*P* = 0.04) and lack of incremental predictive value suggest that this finding requires confirmation in prospective studies.

### Blastocyst transfer: from univariate trend to multivariable analysis

Blastocyst transfer is a commonly used strategy in FET cycles. Blastocysts have more mature morphology and higher implantation potential, theoretically increasing the clinical pregnancy rate. Studies have confirmed that compared with cleavage-stage embryo transfer, blastocyst transfer selects embryos with better developmental potential and improves implantation and clinical pregnancy rates [8, 9, 20].

In this study, univariate analysis showed a trend toward association between blastocyst transfer and clinical pregnancy (OR = 1.59, 95% CI: 0.92–2.75, *P* = 0.10). However, after multivariable adjustment (including age, BMI, ln(β-hCG + 1), and double embryo transfer), blastocyst transfer did not reach statistical significance (OR = 1.39, 95% CI: 0.30–6.32, *P* = 0.67), suggesting that it was not an independent predictor of clinical pregnancy. This finding may be attributable to the limited sample size and potential confounding factors, and warrants further validation in larger studies.

### Negative findings for red blood cell distribution width (RDW)

RDW is an objective indicator of red blood cell volume heterogeneity, traditionally used for anemia differential diagnosis. Recent studies have shown that abnormal RDW is also associated with systemic inflammatory response and oxidative stress. Although some previous studies have suggested that elevated RDW is related to adverse pregnancy outcomes, no significant difference in RDW was observed between the two groups in this study, which may be attributable to sample size, timing of measurement, and the retrospective design.

### Strengths of the study

Strengths of this study include rigorous selection of study subjects and inclusion of all 268 patients (including completely non-pregnant cases) in the ROC analysis, avoiding bias from inclusion of only positive outcomes. In addition, by incorporating globulin-related mechanisms, the study provides an in-depth interpretation of the physiological significance of low TP, addressing the limitation of analyzing total protein and albumin alone. Using ROC curve and multivariable logistic regression analysis, the study identified the predictive value of β-hCG for clinical pregnancy in FET and established an optimal cut-off value, providing a specific reference for clinical practice. It should be noted that blastocyst transfer and TP did not reach statistical significance in multivariable analysis, and these results should be considered exploratory findings.

### Limitations of the study

Several limitations Several limitations should be acknowledged. First, this was a single-center retrospective study with a limited sample size, as reflected by the wide confidence interval for blastocyst transfer (0.30–6.32), requiring validation in larger multicenter prospective cohorts. Second, residual confounding cannot be excluded despite multivariable adjustment. Third, TP showed only marginal significance (*P* = 0.04), and the combined model did not demonstrate significant incremental predictive value; this finding is exploratory and requires confirmation in prospective studies. Fourth, endometrial thickness data were missing for approximately 35% of patients (95/268). A sensitivity analysis limited to patients with available data (*n* = 173) showed largely unchanged results for core variables; however, due to the high missing rate, these results should be interpreted with caution. Fifth, due to the limited follow-up period (up to 12 weeks of gestation), live birth data were not available; future studies with extended follow-up to delivery are needed. Sixth, the biochemical pregnancy subgroup (*n* = 31) was too small to perform a separate ROC analysis. Seventh, ovarian reserve parameters (e.g., antral follicle count, basal FSH, inhibin B) were not comprehensively evaluated. Although AMH levels did not differ significantly between groups (*P* = 0.57), the potential influence of other ovarian reserve parameters cannot be completely excluded [21].

## Conclusions

In this retrospective cohort study, serum β-hCG measured 11–14 days after FET demonstrated excellent predictive performance for clinical pregnancy, with an optimal cut-off value of 112.0 mIU/mL identified in our cohort. Blastocyst transfer showed a trend toward association in univariate analysis but did not reach statistical significance after multivariable adjustment (OR = 1.39, *P* = 0.67). Serum TP level on day 7 post-transfer showed a marginal association in univariate analysis (*P* = 0.04) but did not provide incremental predictive value beyond β-hCG. Alb and RDW showed no significant associations.

These findings suggest that β-hCG has good clinical value for early pregnancy assessment after FET. Due to the limited follow-up period (up to 12 weeks of gestation), live birth data were not available; therefore, clinical pregnancy was used as the primary outcome. External validation in larger, prospective cohorts with extended follow-up to delivery is warranted.

This study has limitations, including a single-center design and a relatively small sample size. Large-sample, multicenter prospective studies are needed to further validate the reliability of these findings. Meanwhile, the development of combined prediction models incorporating multiple indicators to enhance predictive efficacy warrants further exploration.

## Data Availability

The data analyzed in this study is subject to the following licenses/restrictions: The raw data of patients is not publicly available because of patient confidentiality and participant privacy. Requests to access these datasets should be directed to Lijun Peng, Penglijungxy@163.com.

## References

[1] Lensen S, Lantsberg D, Gardner DK, Sophian AD, Wandafiana N, Kamath MS. The role of timing in frozen embryo transfer. Fertil Steril. 2022;118(5):832–8. 10.1016/j.fertnstert.2022.08.009. 36150920

[2] Maheshwari A, Bell JL, Bhide P, et al. Elective freezing of embryos versus fresh embryo transfer in IVF: a multicentre randomized controlled trial in the UK (E-Freeze). Hum Reprod. 2022;37(3):476–87. 10.1093/humrep/deab279. 34999830 PMC9206534

[3] Wang L, Jiang Y, Shen H, et al. Independent value of serum β-human chorionic gonadotropin in predicting early pregnancy loss risks in IVF/ICSI cycles. Front Immunol. 2022;13:992121. 10.3389/fimmu.2022.992121. Published 2022 Sep 29. 36248885 PMC9556765

[4] Bollig KJ, Senapati S, Sammel MD, et al. Validation of a multiple marker test for early pregnancy outcome prediction. J Assist Reprod Genet. 2023;40(4):837–44. 10.1007/s10815-023-02719-w. 36708430 PMC10224881

[5] Davari Tanha F, Rasti A, Pakniat H, Salimi Setudeh S, Hosseini A, Rahimian Ghohroodi MA. Serum β-hCG, AMH, TSH, and PRL Levels in Predicting the Outcome of Early Pregnancy 14 Days after Embryo Transfer: A Prospective Cohort Study. Adv Biomed Res. 2025;14:45. 10.4103/abr.abr_285_23. Published 2025 May 31. 40519571 PMC12165297

[6] Gao W, Tang W, Li C, et al. Effect of maternal age, embryo number and quality on pregnancy outcome during frozen embryo transfer cycle. Front Endocrinol (Lausanne). 2025;16:1596178. 10.3389/fendo.2025.1596178. Published 2025 Nov 7. 41282292 PMC12634337

[7] Kalafat E, Ata B, Del Gallego R, et al. Predicting live birth following single euploid frozen-embryo transfer via β-hCG dynamics: iHOPE prognostic model with external validation. Ultrasound Obstet Gynecol. 2026;67(6):824–35. 10.1002/uog.70238. 42177810

[8] Jiang J, Wu X, Sun H, et al. Effects of Various Transfer Strategies of Frozen-Thawed Cleavage-Stage and Blastocyst Embryos on Pregnancy and Neonatal Outcomes in Different Age Groups. J Multidiscip Healthc. 2025;18:2319–34. 10.2147/JMDH.S502766. 40302817 PMC12039847

[9] Chang CT, Weng SF, Chuang HY, Hsu IL, Hsu CY, Tsai EM. Embryo transfer impact: a comprehensive national cohort analysis comparing maternal and neonatal outcomes across varied embryo stages in fresh and frozen transfers. Front Endocrinol (Lausanne). 2024;15:1400255. 10.3389/fendo.2024.1400255. Published 2024 Jun 12. 38933826 PMC11199782

[10] Chin KTC, Yiu JHC, Cheung KW, et al. Flagellin in blood and Bifidobacteriumpseudocatenulatum in gut are associated with live birth upon IVF-frozen embryo transfer. iScience. 2025;28(2):111933. 10.1016/j.isci.2025.111933. Published 2025 Jan 31. 40034118 PMC11872633

[11] Li J, Jiang X, Li C, Che H, Ling L, Wei Z. Proteomic alteration of endometrial tissues during secretion in polycystic ovary syndrome may affect endometrial receptivity. Clin Proteom. 2022;19(1). 10.1186/s12014-022-09353-1. 19.Published 2022 May 28. PMC9145147 35643455

[12] Mourad A, Antaki R, Rowen M, Lévesque É, Lapensée L. The POPI-Plus tool: prediction model of outcome of pregnancy in in vitro fertilization from a large retrospective cohort. Fertil Steril. 2024;121(3):489–96. 10.1016/j.fertnstert.2023.11.035. 38043845

[13] Wang Tao Z, Huina W, Zhongwei, et al. Correlation between serum β-HCG level on day 14 after frozen-thawed embryo transfer and live birth rate and its predictive value[J]. J Reprod Med. 2023;32(2):181–7.

[14] Qiu P, Wang Y, Ji H, et al. Predictive value of serum HCG concentrations for outcomes of vitrified-warmed blastocyst transfers in women of different ages. Reprod Biomed Online. 2021;43(5):962–9. 10.1016/j.rbmo.2021.07.004. 34565674

[15] de la Cruz R, Lavielle M, Meza C, Núñez-Antón V. A joint analysis proposal of nonlinear longitudinal and time-to-event right-, interval-censored data for modeling pregnancy miscarriage. Comput Biol Med. 2024;182:109186. 10.1016/j.compbiomed.2024.109186. 39362003

[16] Hernandez-Nieto C, Lee J, Alkon-Meadows T, et al. Biological relevance of trophectoderm morphology: initial β-hCG measurements correlate with trophectoderm grading on euploid frozen embryo transfers. J Assist Reprod Genet. 2022;39(9):2051–9. 10.1007/s10815-022-02553-6. 35751829 PMC9475011

[17] Wu M, Xiao X, Wang C, et al. Combined analysis of estradiol and β-hCG to predict the early pregnancy outcome of FET: a retrospective study. J Ovarian Res. 2024;17(1):128. 10.1186/s13048-024-01433-0. Published 2024 Jun 21. 38907300 PMC11193290

[18] Wang L, Jiang Y, Shen H, et al. Independent value of serum β-human chorionic gonadotropin in predicting early pregnancy loss risks in IVF/ICSI cycles. Front Immunol. 2022;13:992121. 10.3389/fimmu.2022.992121. Published 2022 Sep 29. 36248885 PMC9556765

[19] Rizzuto G. B Cell Responses to the Placenta and Fetus. Annu Rev Pathol. 2025;20(1):33–58. 10.1146/annurev-pathmechdis-111523-023459. 39264989 PMC11912550

[20] Glujovsky D, Quinteiro Retamar AM, Alvarez Sedo CR, Ciapponi A, Cornelisse S, Blake D. Cleavage-stage versus blastocyst-stage embryo transfer in assisted reproductive technology. Cochrane Database Syst Rev. 2022;5(5):CD002118. 10.1002/14651858.CD002118.pub6. Published 2022 May 19. 35588094 PMC9119424

[21] Aykanat G, Ertan B, Kalkan FT, et al. Association between menstrual cycle length and ovarian reserve markers and stimulation response: a retrospective cohort study. BMC Women‘s Health. 2026;26:159. 10.1186/s12905-026-04391-5. 41803788 PMC12980889

